# Supplementary material for: Spatiotemporal dynamics characterise spectral connectivity profiles of continuous speaking and listening
Source: PLoS Biol. 2023 Jul 21;21(7):e3002178. doi: 10.1371/journal.pbio.3002178 (PMC12716320; doi:10.1371/journal.pbio.3002178)
Supplement: S1 Table — (DOCX) [file pbio.3002178.s001.docx]

| **Speech production** | **Speech production with masked perception** |
| --- | --- |
| What are your plans for today and the coming days? | Describe a popular artist / author / regisseur. What makes them interesting to you? |
| Which animals do you like? | Describe a traditional christmas. |
| Which hobbies do / did you have? | Where would you like to go on vacation? |
| What does a typical weekend look like for you? | Which places do you like to go in Münster and what is there to see / do? |
| What types of food do you like? | Describe what happens during the olympic games. |
| Describe a popular singer / musician / composer. What do you like about them? | Describe the geography and nature of Africa. |
| Which movie-, literature-, comic-character would you like to be and why? | What comes to your mind when you think of poker? |

**S1 Table.** Full list of questions participants had to answer in the first (speech production) and second recording (speech production with masked perception).
