# Supplementary material for: Spatiotemporal dynamics characterise spectral connectivity profiles of continuous speaking and listening
Source: PLoS Biol. 2023 Jul 21;21(7):e3002178. doi: 10.1371/journal.pbio.3002178 (PMC12716320; doi:10.1371/journal.pbio.3002178)
Supplement: S1 Fig — Cortical map represents groups statistics of multivariate MI between speech envelope and brain activity in each parcel compared to 95th percentile of surrogate data. Colour code represents the sum of all significant T-values across delay and frequency (FDR-corrected across delays (−1 to 1 s), frequencies (1 to 10 Hz), and parcels). The data underlying this figure can be found in https://osf.io/9fq47/. (DOCX) [file pbio.3002178.s002.docx]

**
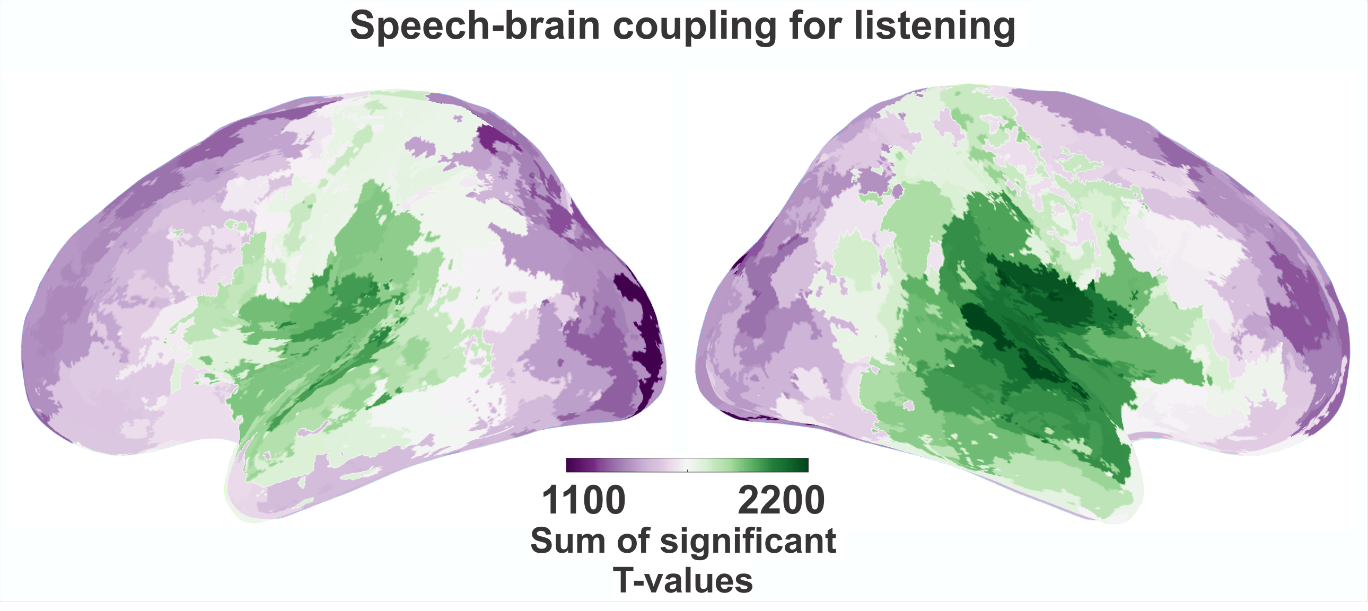
**

**S1 Fig. Results of speech brain coupling for the listening condition.** Cortical map represents groups statistics of multivariate MI between speech envelope and brain activity in each parcel compared to 95th percentile of surrogate data. Colour code represents the sum of all significant T-values across delay and frequency (FDR-corrected across delays (-1-1 s), frequencies (1 to 10 Hz) and parcels). The data underlying this Figure can be found in https://osf.io/9fq47/.
