# Supplementary material for: Spatiotemporal dynamics characterise spectral connectivity profiles of continuous speaking and listening
Source: PLoS Biol. 2023 Jul 21;21(7):e3002178. doi: 10.1371/journal.pbio.3002178 (PMC12716320; doi:10.1371/journal.pbio.3002178)
Supplement: S2 Fig — Results were spatially interpolated to the HCP atlas used in our study. Colour codes correspond to z-values from a uniformity test as documented on neurosynth.org. Lower panel: The relative difference of correlations (r1-r2)/(r1+r2) across delays. r1 is the correlation between our speech production network and the neurosynth speech production network and r2 is the correlation between our speech production network and the neurosynth speech perception network. The data underlying this figure can be found in https://osf.io/9fq47/. (DOCX) [file pbio.3002178.s003.docx]

*Comparison of our speech production network with fMRI meta-analysis*

To assess the localisation accuracy of our MEG speech production network based on speech-brain coupling we compared our results to the automatic meta analysis provided by neurosynth.org. Using the term ‘speech production’ resulted in a meta analysis of 107 fMRI studies investigating speech production. We downloaded the statistical meta analysis map that corresponded to a uniformity test. The map of z-scores results ‘from a one-way Anova testing whether the proportion of studies that report activation at a given voxel differs from the rate that would be expected if activations were uniformly distributed throughout gray matter’ (quote from neurosynth.org). The map therefore corresponds to a classical fMRI statistical analysis and measures consistency of activations across studies. This volumetric statistical map was then transformed to the HCP atlas in the following way: First, we interpolated the statistical map to the surface representation of the HCP atlas using ft_sourceinterpolate.m in fieldtrip. Next, we averaged all statistical values within the same anatomical parcel. This resulted in one value per parcel that could be directly compared to the statistical maps from our study (which are also based on one value per parcel). Both statistical maps represent a measure of consistency (across studies for neurosynth and across participants for our study) and can be expected to be similar. We therefore hypothesised that parcels with high statistical values in the fMRI meta analysis (neurosynth) also show high statistical values in our study (and correspondingly for parcels with low values). We therefore correlated the statistical values of both statistical maps across parcels. The correlation was highly significant (r=0.42, p<<0.0001). To further test significance we performed a permutation test and computed the correlation 1000 times on randomly permuted values which resulted in a 99th percentile of this null distribution of r_0_=0.16. Both results demonstrate a highly significant relationship between both statistical maps and indicate that our results are valid.


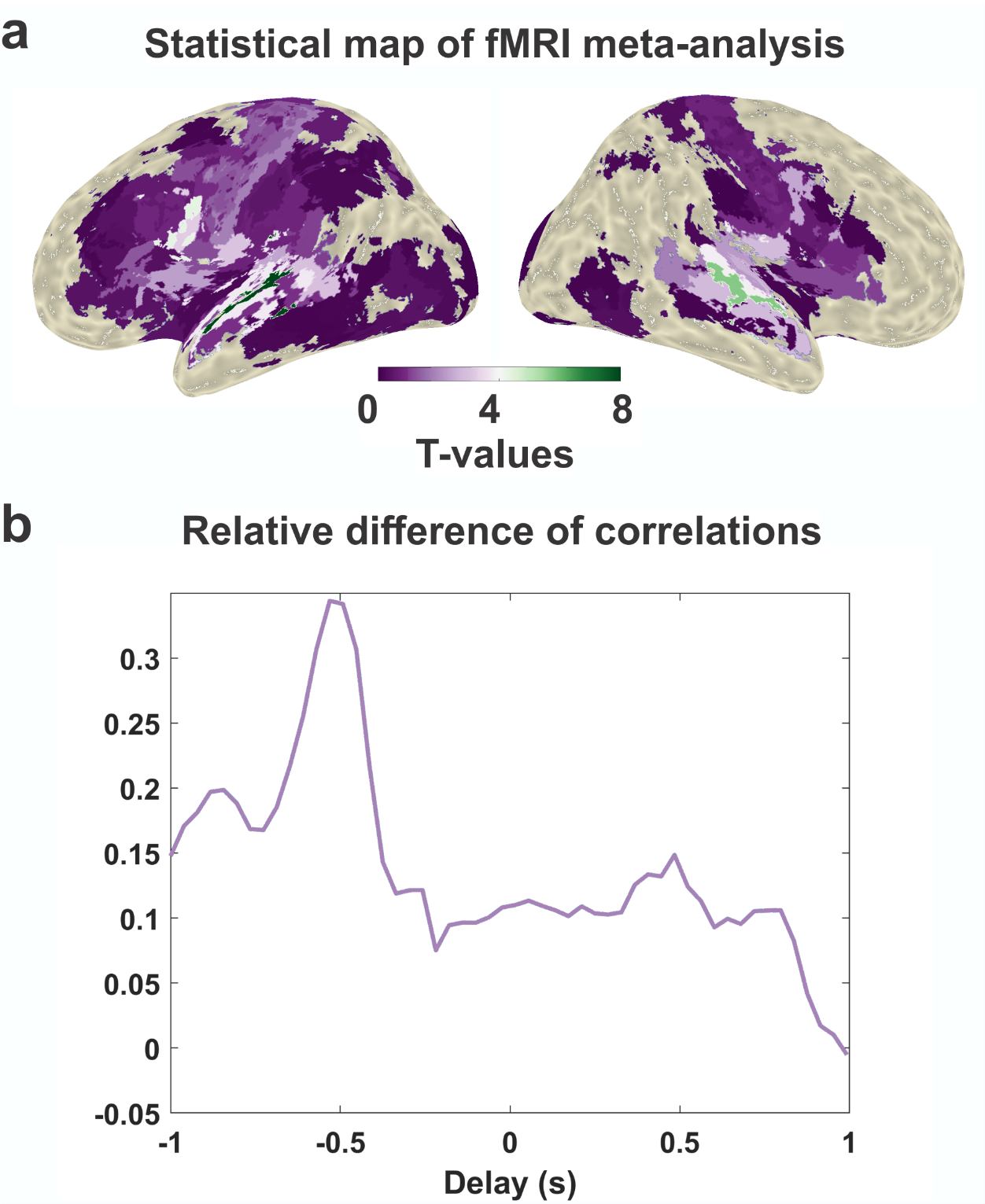


**S2 Fig** Upper panel**:** Statistical map of fMRI meta-analysis from neurosynth.org using the search term ‘speech production’**.** Results were spatially interpolated to the HCP atlas used in our study. Color codes correspond to z-values from a uniformity test as documented on neurosynth.org. Lower Panel: The relative difference of correlations (r1-r2)/(r1+r2) across delays. r1 is the correlation between our speech production network and the neurosynth speech production network and r2 is the correlation between our speech production network and the neurosynth speech perception network. The data underlying this Figure can be found in https://osf.io/9fq47/.

Still, one could argue that correlations between our statistical map and the speech production meta analysis from neurosynth.org might be high and significant for any network and therefore not specific for the speech production network. Therefore, we repeated the analysis for three other networks: motor network, resting-state network and speech perception network (the names used here also represent the neurosynth search terms). We like to note that including the speech perception network represents a formidable challenge since it shows a large overlap with the speech production network. As before, we correlated these three additional maps with our speech production map (Fig. 2) and observed the following correlations (speech perception r=0.33, p<<0.001; motor network r=0.18, p=0.007, resting state r=-0.07, p>0.05, all df=228). Therefore, our speech production network most closely resembles the speech production network from neurosynth. To test how robustly the correlation with the neurosynth speech production network is higher than the neurosynth speech perception network we performed 10000 bootstrap correlations (with random selection of 230 parcels with replacement in both statistical maps) and counted how often the correlation with the speech production network was higher than the correlation with the speech perception network. This was the case in 99.7% of the bootstrap iteration indicating that our network robustly resembles the neurosynth speech production network better than the neurosynth speech perception network.

Finally, in another stringent test of the validity of our results we tested the hypothesis that the higher correlation of our speech production network to the neurosynth speech production network compared to the neurosynth speech perception network is delay dependent. Specifically, the correlation should be highest for negative delays (where brain activity precedes the speech envelope. The lower panel of Suppl. Fig. 2 shows the relative difference of correlations (r1-r2)/(r1+r2) across delays. r1 is the correlation between our speech production network and the neurosynth speech production network and r2 is the correlation between our speech production network and the neurosynth speech perception network. A clear positive peak is evident at negative lags indicates that the best match between speech production networks in neurosynth and in our analysis occur for brain activity preceding the speech envelope.
