# Supplementary material for: Spatiotemporal dynamics characterise spectral connectivity profiles of continuous speaking and listening
Source: PLoS Biol. 2023 Jul 21;21(7):e3002178. doi: 10.1371/journal.pbio.3002178 (PMC12716320; doi:10.1371/journal.pbio.3002178)
Supplement: S3 Fig — The data underlying this figure can be found in https://osf.io/9fq47/. (DOCX) [file pbio.3002178.s004.docx]

*Lateralisation (Right vs left) for listening*


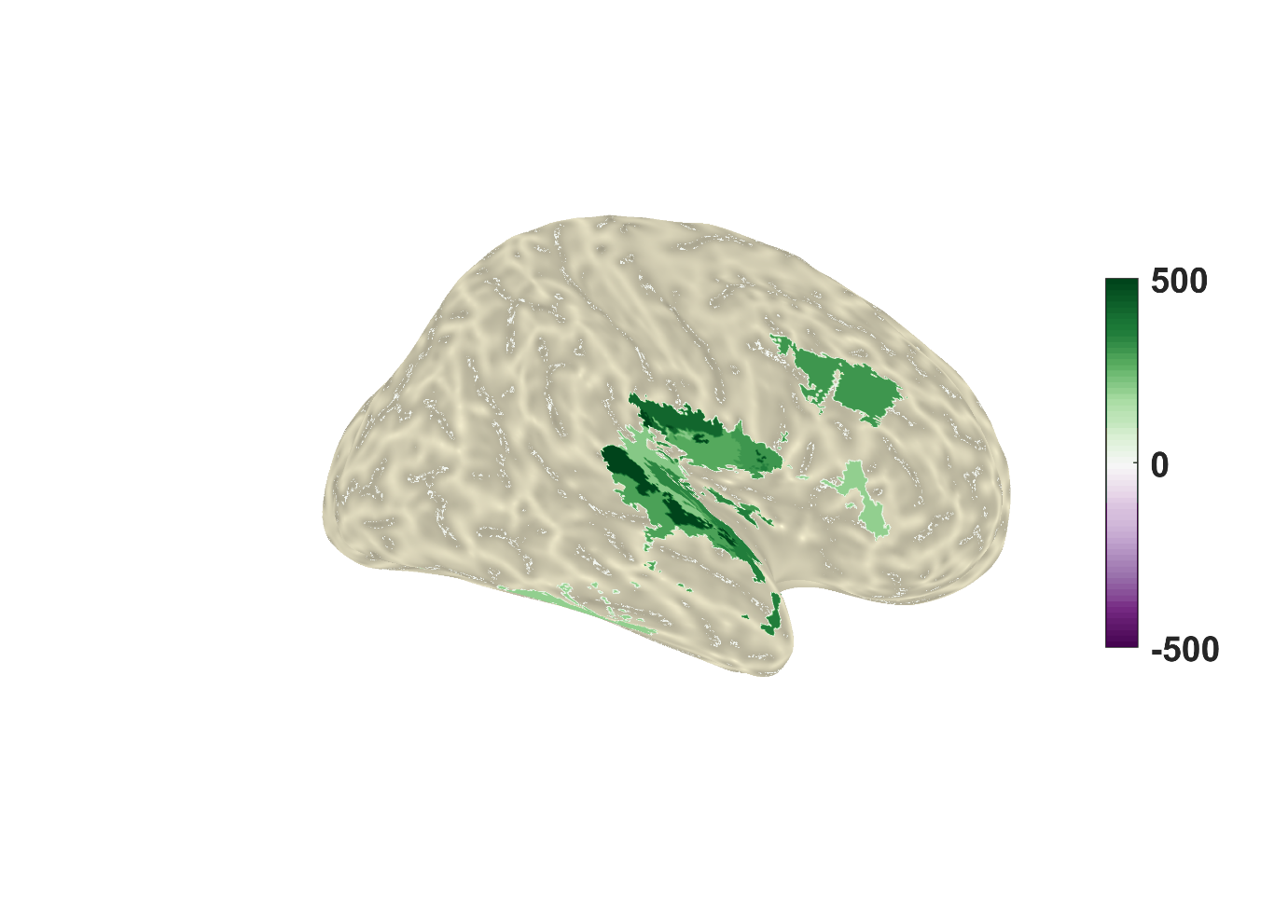


**S3 Fig. Speech-brain coupling lateralisation for listening condition.** The data underlying this Figure can be found in https://osf.io/9fq47/.
