# Supplementary material for: Spatiotemporal dynamics characterise spectral connectivity profiles of continuous speaking and listening
Source: PLoS Biol. 2023 Jul 21;21(7):e3002178. doi: 10.1371/journal.pbio.3002178 (PMC12716320; doi:10.1371/journal.pbio.3002178)
Supplement: S4 Fig — Colour codes t-values. The data underlying this figure can be found in https://osf.io/9fq47/. (DOCX) [file pbio.3002178.s005.docx]

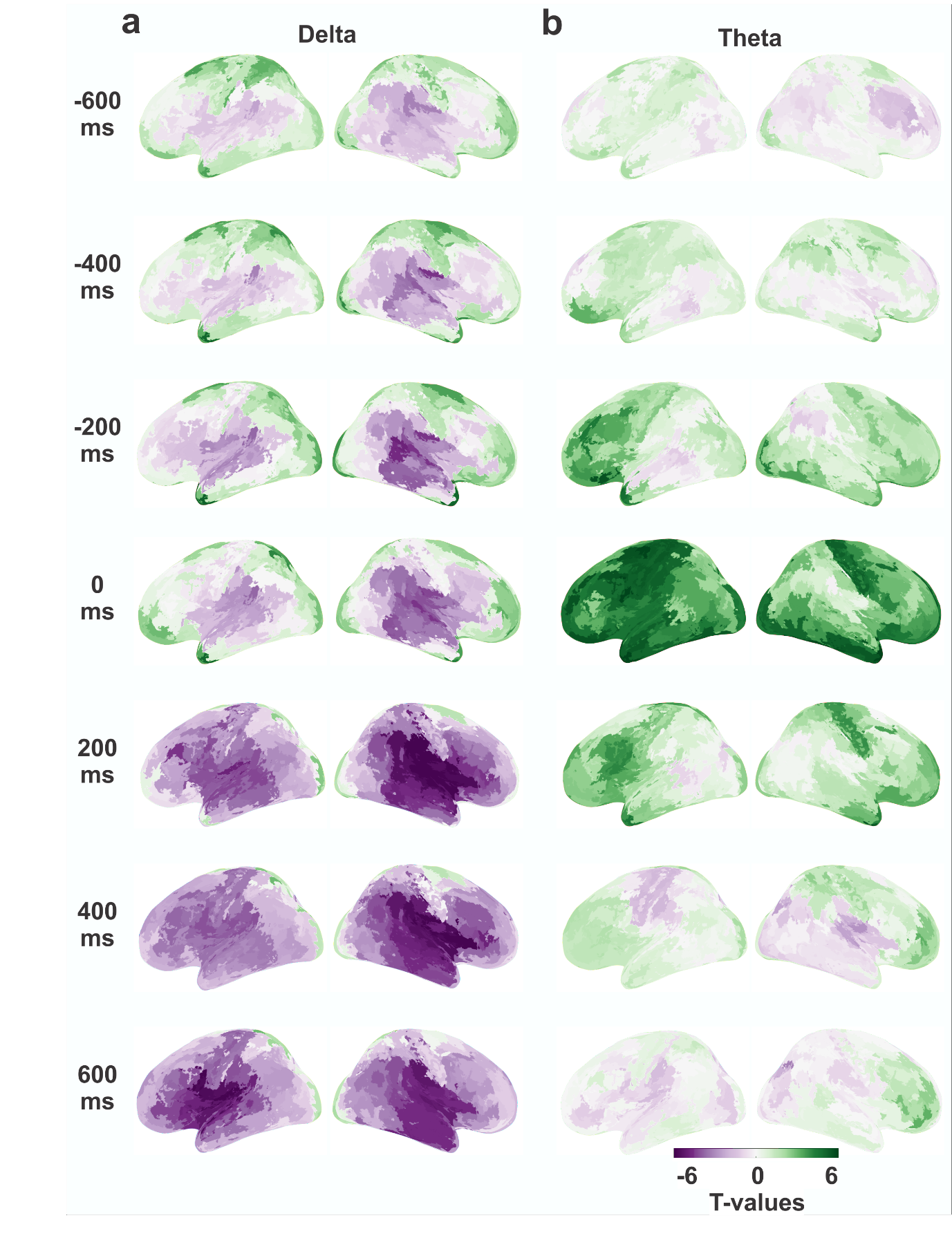


**S4 Fig.** Statistical comparison of speech-brain coupling in speaking versus listening at delta (2 Hz) and theta (5 Hz) for different delays ([-600 600] ms). Colour codes t-values. The data underlying this Figure can be found in https://osf.io/9fq47/.
