# Supplementary material for: Spatiotemporal dynamics characterise spectral connectivity profiles of continuous speaking and listening
Source: PLoS Biol. 2023 Jul 21;21(7):e3002178. doi: 10.1371/journal.pbio.3002178 (PMC12716320; doi:10.1371/journal.pbio.3002178)
Supplement: S6 Fig — Significant connectivity between STG and other cortical parcels during speech production (a) and listening (b) in different frequency bands. A cluster-based permutation test was used to detect significant connectivity patterns (p < 0.05). Colour codes t-values. Purple colour represents the flow of information from STG to other cortical parcels and the green colour represents the opposite direction. The data underlying this figure can be found in https://osf.io/9fq47/. (DOCX) [file pbio.3002178.s007.docx]

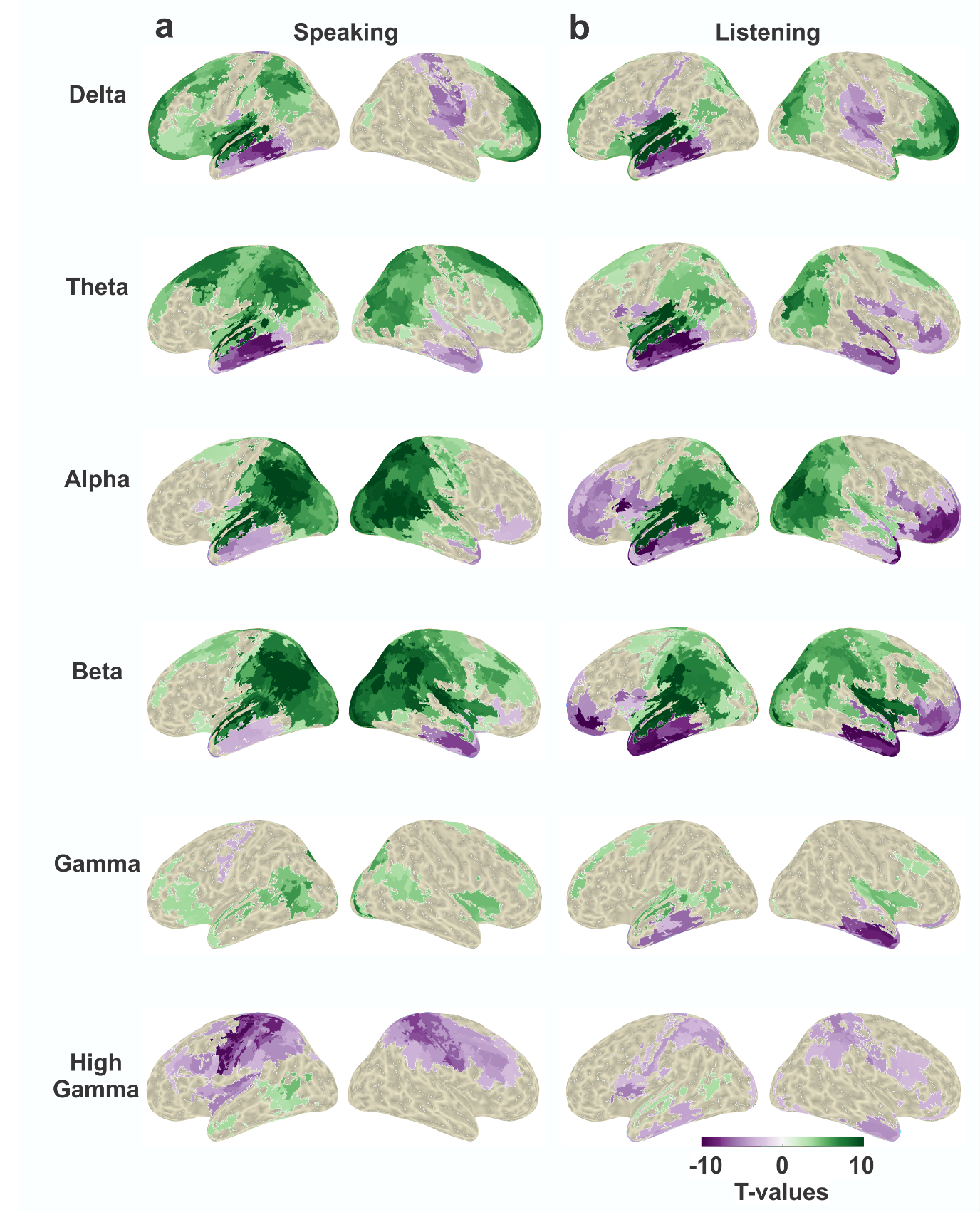


**S6 Fig.** Significant connectivity between STG and other cortical parcels during speech production (a) and listening (b) in different frequency bands. A cluster-based permutation test was used to detect significant connectivity patterns (p<0.05). Colour codes t values. Purple colour represents the flow of information from STG to other cortical parcels and the green colour represents the opposite direction. The data underlying this Figure can be found in https://osf.io/9fq47/.
