# Supplementary material for: Spatiotemporal dynamics characterise spectral connectivity profiles of continuous speaking and listening
Source: PLoS Biol. 2023 Jul 21;21(7):e3002178. doi: 10.1371/journal.pbio.3002178 (PMC12716320; doi:10.1371/journal.pbio.3002178)
Supplement: S7 Fig — A cluster-based permutation test was used to detect significant connectivity differences from all the cortical parcels to STG between speaking and listening conditions (p < 0.05). Colour codes t-values. Please note that since we compared asymmetry indices (DAI) between 2 conditions, interpreting the directionality from this cortical plot is challenging. For a better understanding of these statistical maps, please refer to the spectrally resolved DAI between STG and 4 ROIs in Fig 7D as well as S3 Fig. The data underlying this figure can be found in https://osf.io/9fq47/. (DOCX) [file pbio.3002178.s008.docx]

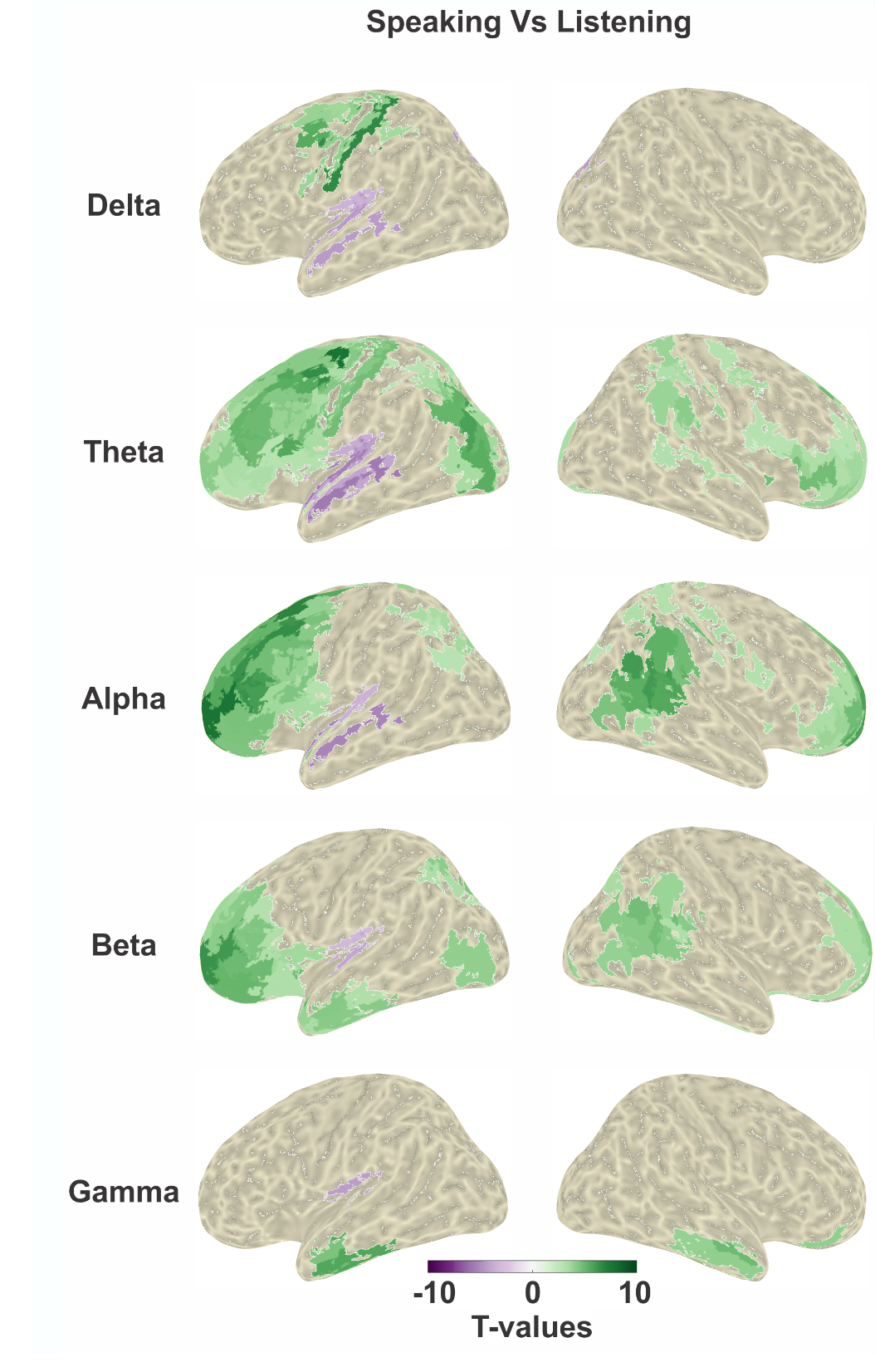


**S7 Fig.** Comparison of connectivity patterns between speaking and listening conditions in different frequency bands. A cluster-based permutation test was used to detect significant connectivity differences from all the cortical parcels to STG between speaking and listening conditions (p<0.05). Colour codes t values. Please note that since we compared asymmetry indices (DAI) between two conditions, interpreting the directionality from this cortical plot is challenging. For a better understanding of these statistical maps, please refer to the spectrally-resolved DAI between STG and four ROIs in Fig. 7d as well as supplementary Fig. 3. The data underlying this Figure can be found in https://osf.io/9fq47/.
