# Supplementary material for: Spatiotemporal dynamics characterise spectral connectivity profiles of continuous speaking and listening
Source: PLoS Biol. 2023 Jul 21;21(7):e3002178. doi: 10.1371/journal.pbio.3002178 (PMC12716320; doi:10.1371/journal.pbio.3002178)
Supplement: S8 Fig — Speech-STG coupling in theta range (positively lagged: 130 ms) is negatively correlated with the top-down delta (a) as well as theta (b) connectivity from mainly left occipital and parietal areas. The data underlying this figure can be found in https://osf.io/9fq47/. (DOCX) [file pbio.3002178.s009.docx]

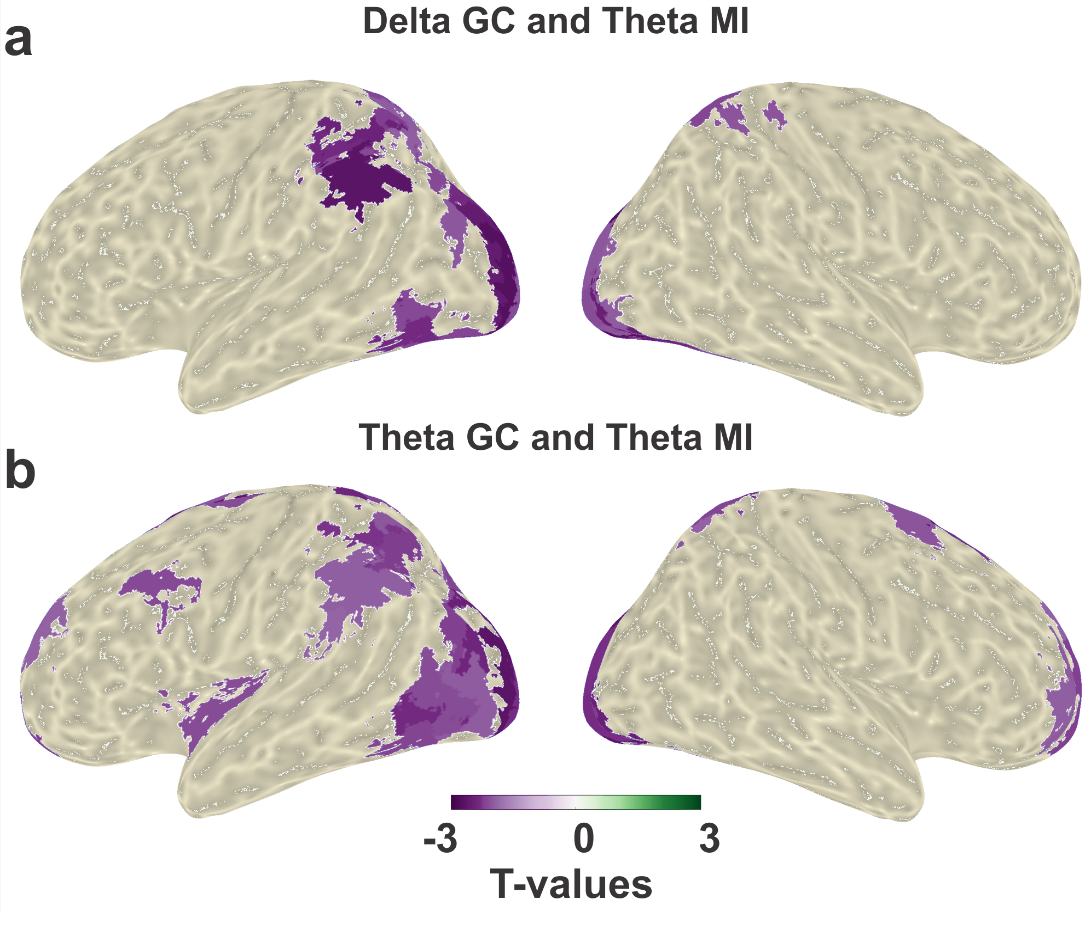


**S8 Fig. Correlation analysis between top-down GC and MI.** Speech-STG coupling in theta range (positively lagged: 130ms) is negatively correlated with the top-down delta (a) as well as theta (b) connectivity from mainly left occipital and parietal areas. The data underlying this Figure can be found in https://osf.io/9fq47/.
