# Supplementary material for: Spatiotemporal dynamics characterise spectral connectivity profiles of continuous speaking and listening
Source: PLoS Biol. 2023 Jul 21;21(7):e3002178. doi: 10.1371/journal.pbio.3002178 (PMC12716320; doi:10.1371/journal.pbio.3002178)
Supplement: S9 Fig — (a) The statistical contrast of frequency-specific power between speaking and listening. Results of dependent-samples t test were FDR corrected across parcels and frequencies (1–100 Hz). Colour codes sum of t-values across all significant frequencies. Note that we use a one-tailed test (speaking < listening) to ensure that results are not contaminated by residual speech artefacts. (b) Power spectral density averaged across all significant parcels for speaking (blue) and listening (red) conditions. The grey panel depicts the frequency range 10–30 Hz. The data underlying this figure can be found in https://osf.io/9fq47/. (DOCX) [file pbio.3002178.s010.docx]

*Statistical contrast of frequency-specific power between speaking and listening*

We also calculated the statistical contrast of frequency-specific power (not coupling to the speech envelope) between speaking and listening. The analysis reveals significant positive and negative effects. However, positive effects (higher power in speaking compared to listening) could be (partly) caused by residual speech artefacts (such as muscle artefacts or movement). Therefore, in Supplementary Fig. 1 we report only effects where power in any frequency band is significantly lower in the speaking condition compared to listening. As expected, we see the strongest power decrease (during speaking compared to listening) over bilateral motor areas extending to premotor and parietal areas with a dominance in the left hemisphere. The power suppression in motor and premotor areas includes medial supplementary motor areas (SMA) and extends all the way down to lateral motor areas corresponding to speech-related motor representations of larynx, tongue, and mouth. Supplementary Fig. 1b shows that these suppression effects arise from power differences at frequencies between about 10 Hz and 30 Hz, largely corresponding to the beta frequency band that has been consistently related to motor functions [(](https://sciwheel.com/work/citation?ids=337955,222896&pre=&pre=&suf=&suf=&sa=0,0)1, 2[)](https://sciwheel.com/work/citation?ids=337955,222896&pre=&pre=&suf=&suf=&sa=0,0).


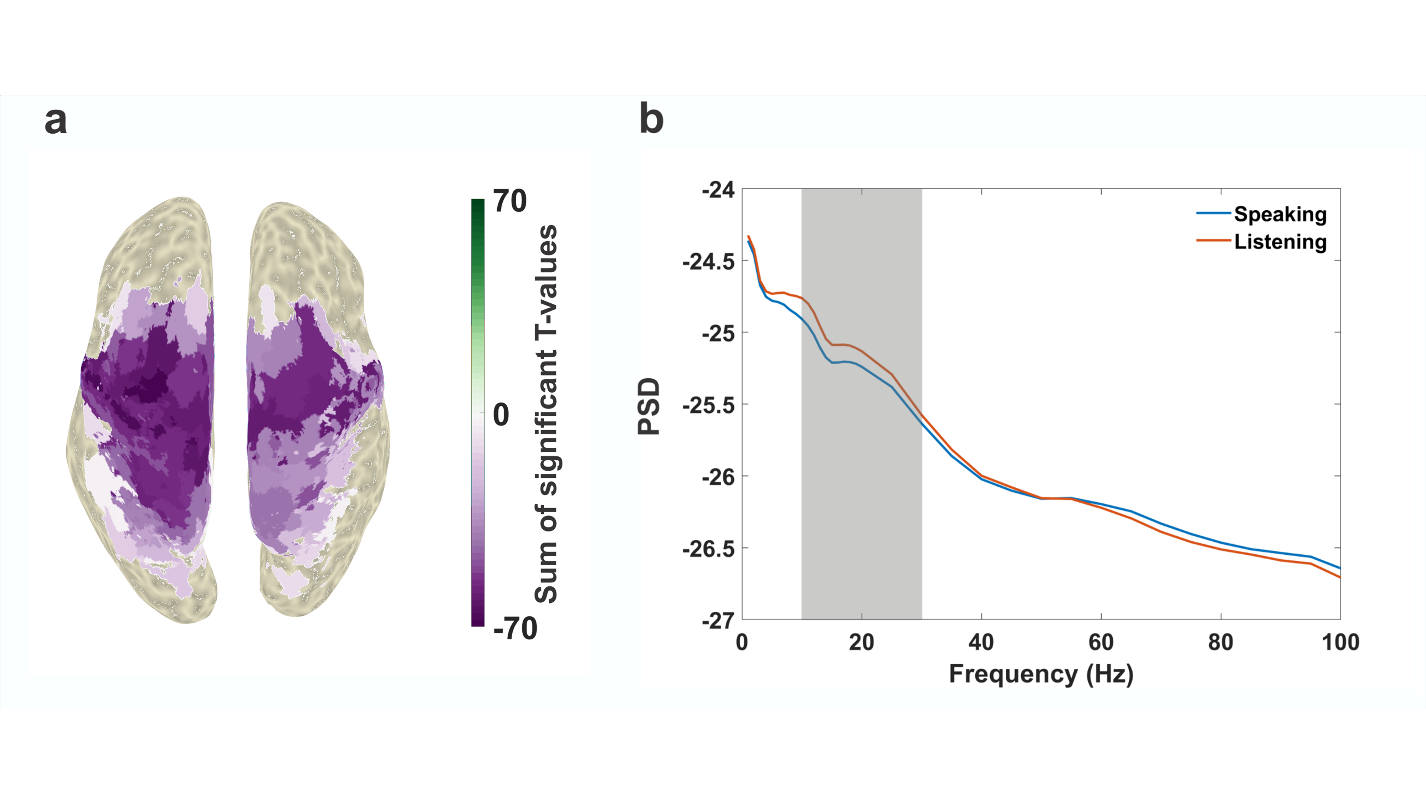


**S9 Fig. Frequency-specific power comparison between speaking and listening condition. a.** The statistical contrast of frequency-specific power between speaking and listening. Results of dependent-samples t-test were FDR corrected across parcels and frequencies (1-100 Hz). Colour codes sum of t-values across all significant frequencies. Note that we use a one-tailed test (speaking < listening) to ensure that results are not contaminated by residual speech artefacts. **b.** Power spectral density averaged across all significant parcels for speaking (blue) and listening (red) conditions. The grey panel depicts the frequency range 10-30 Hz. The data underlying this Figure can be found in https://osf.io/9fq47/.

Reference

1. Jenkinson N, Brown P. New insights into the relationship between dopamine, beta oscillations and motor function. Trends Neurosci. 2011 Dec;34(12):611–8.

2. Engel AK, Fries P. Beta-band oscillations--signalling the status quo? Curr Opin Neurobiol. 2010 Apr;20(2):156–65.
