# Supplementary material for: Spatiotemporal dynamics characterise spectral connectivity profiles of continuous speaking and listening
Source: PLoS Biol. 2023 Jul 21;21(7):e3002178. doi: 10.1371/journal.pbio.3002178 (PMC12716320; doi:10.1371/journal.pbio.3002178)
Supplement: Attachment — Submitted filename: Response to reviewer PLOS Biology.docx [file pbio.3002178.s011.docx]

# **Response to Reviewers (PBIOLOGY-D-23-00327R1):**

We would like to thank our two reviewers for their remaining positive comments, which helped us to further improve our manuscript. We have carefully revised the manuscript to integrate the reviewer’s comments and suggestions.

Below, we address the reviewer’s comments point-by-point.

**Changes in the manuscript are marked in red.**

**Reviewer #2:**

I reviewed a previous version of the manuscript for another journal. My current review addresses the comments raised by all the previous reviewers. As I previously wrote, this work is innovative, methodologically and conceptually, the methods are sound, and the results are important to further our understanding of the brain as being a dynamical system.

Concerning the previous concerns of Reviewer #1, I tend to agree with their, and appreciate that the authors have deemphasized references to predictive coding from their manuscript.

However, I would suggest removing any mention of predictive coding in the abstract (where it is present in two instances). Indeed, none of the results investigate predictive processing per se, as is for instance done with the estimation of surprisal or entropy variables that are then correlated to brain dynamics and connectivity patterns. Here predictive coding is only one of the possible interpretations of their result (and not the most straightforward).

We are grateful for the valuable feedback provided by the reviewer during all stages of the reviewing process. We agree with the reviewer and removed the ‘predictive coding’ term from the abstract and now have it as one of the possible interpretations of our results in the discussion.

The authors also added a fifth question in the introduction, but again I would use caution regarding this point (and actually, I suggest to simply remove it), for a specific set of related reasons:

-	First, beta (∼20 Hz) activity is the default oscillatory mode of the motor system.

-	Second, the framework championed by A. Bastos and colleagues (that the authors rely on here) has recently been heavily put into question, notably by the work of M. Vinck and colleagues (see, e.g. Schneider et al., Neuron 2021, or Vinck Neuron 2023 - but also by the group of C.E. Schroeder or A. Thiele). They basically show that inter-areal directed coherence can be predicted by the dominant power amongst the two explored regions.

-	Third, regrouping these two information results in the hypothesis that beta activity will be necessarily be directed from motor to auditory regions. Imho, this reflects the most parsimonious hypothesis, and there is no need to rely on predictive coding assumptions of dedicated frequency channels.

-	Finally, as I said before, the authors entirely rely on the proposal by Bastos et al. (which was actually never empirically validated!) but do not show any evidence that some predictions or prediction error signals are transmitted between motor and auditory regions in the beta band. It is simply an interpretation of their results that they can of course mention in the discussion. But I would use caution (and mention alternative interpretations too, such as the one described above, for instance).

We thank the reviewer for raising this point. We fully agree with the reviewer and removed the fifth question from the manuscript. Now in the new version of the manuscript we interpreted our results with more caution and also added the recent papers that were suggested by the reviewer in our discussion.

*These findings suggest a potential alignment with the ‘predictive coding’ framework that would interpret the top-down signals as predictions and bottom-up signals as prediction errors. Nevertheless, it is important to proceed with caution before drawing definitive conclusions since recent studies have raised concerns about the idea that bottom-up and top-down signals are communicated via distinct frequency channels (Schneider et al. 2021; Vinck et al. 2023; Ferro et al. 2021). Therefore, further investigation is needed to fully establish the extent of compatibility between our results and the predictive coding framework. (p. 19)*

Regarding the negative-lag result, I would also first emphasize the most accepted interpretations, notably that the motor cortex is involved in motor preparation, then in the processing of auditory feedback (see, e.g. Ozker…Flinker, PloS Biol 2022 for a relevant example), and then in predictive processing.

We fully agree with the reviewer that further interpretation is needed for this part of our results. In the new version of our manuscript, we added the additional interpretation:

*This coupling at negative lags most likely suggests the involvement of motor preparations or the representation of motor signals involved in controlling speech articulators. Another explanation for the observed negative lags coupling in the motor area could be due to the processing of auditory feedback* [*(Ozker et al. 2022)*](https://sciwheel.com/work/citation?ids=12403081&pre=&suf=&sa=0)*. Furthermore, the detection of negative lag coupling extending beyond the primary motor cortex lends support to the notion of predicting the sensory consequences of expected speech output before they are actually generated* [*(Hickok 2012; Bourguignon et al. 2020; Houde and Chang 2015; Pérez et al. 2022)*](https://sciwheel.com/work/citation?ids=3264251,8821735,8966023,14831372&pre=&pre=&pre=&pre=&suf=&suf=&suf=&suf=&sa=0,0,0,0)*.* (p. 18)

I have no further comments and congratulate the authors for their excellent work.

**Reviewer #3:**

The study's main contribution is a comprehensive description of the brain tracking of the speech envelope during continuous speaking and listening. Moreover, it also describes connectivity patterns that could be interpreted in the light of the predictive coding framework.

Since a substantial part is dedicated to the understanding of the neural substrate of speaking, the inclusion of relevant related bibliography could strengthen the arguments and provide additional context to the findings:

Pérez, A. et al. Timing of brain entrainment to the speech envelope during speaking, listening and self-listening. Cognition (2022).

Ozker M. et al. A cortical network processes auditory error signals during human speech production to maintain fluency. Plos Biology (2022).

We thank the reviewer for the ongoing positive evaluation of our manuscript. We added the two suggested relevant studies in the new version of our manuscript.

Page 5 of the document: the narrative ends abruptly with the disconnected paragraph "Recently, we characterised …".

We fully agree with the reviewer regarding the need for better coherence between the two paragraphs. In response, we have included a sentence to facilitate a smoother transition and improve the flow of the introduction:

*In order to use MEG/EEG approaches for studying speech production, removing the induced speech-related movement artifact is crucial. Recently, we characterised the effects of head movement on the recorded MEG data during speech production (41). In the same study, we also suggested an artefact rejection approach, based on regression analysis and signal space projection (SSP), to remove the induced head movement artefacts from the recorded MEG data and make the current study feasible. (p. 5)*

To improve clarity, the captions of Figures 3 and 4 should include "during speaking".

As the reviewer suggested, we have added this information to the caption of figures 3 and 4 in the new version of the manuscript.
